# Delayed rupture of a pseudoaneurysm in the brachial artery of a burn reconstruction patient

**DOI:** 10.1186/1749-7922-8-21

**Published:** 2013-06-13

**Authors:** Jun Yong Lee, Hyeri Kim, Ho Kwon, Sung-No Jung

**Affiliations:** 1 Department of Plastic and Reconstructive Surgery, Uijeongbu St. Mary’s Hospital, The Catholic University of Korea, 271 Cheonbo-ro, Uijeongbu-si, Gyeonggi-do 480-717, Korea

**Keywords:** Pseudoaneurysm, Brachial artery, Burn, Reconstruction

## Abstract

A brachial artery pseudoaneurysm is a rare but serious condition that can be limb threatening. A number of reports have found that it may be the result of damage to the blood vessels around the brachial artery, either directly or indirectly, due to trauma or systemic diseases. We present our experience of delayed pseudoaneurysm rupture of the brachial artery in a rehabilitation patient with burns of the upper extremity who underwent fasciotomy and musculocutaneous flap coverage. We also provide a review of the brachial artery pseudoaneurysm.

## Introduction

A pseudoaneurysm of the peripheral artery is very rare and is generally a late sequela of trauma, iatrogenic injury, and general illness. It is more infrequent in the upper limb vasculature than in the lower limb vasculature. Although there are many reported causes of brachial artery pseudoaneurysms, to our knowledge, this is the first report of delayed rupture of a brachial artery pseudoaneurysm during the rehabilitation of a patient with burns of the upper extremity who underwent fasciotomy and musculocutaneous flap coverage. We also present a review of the brachial artery pseudoaneurysm.

## Presentation of case

**Figure 1:**
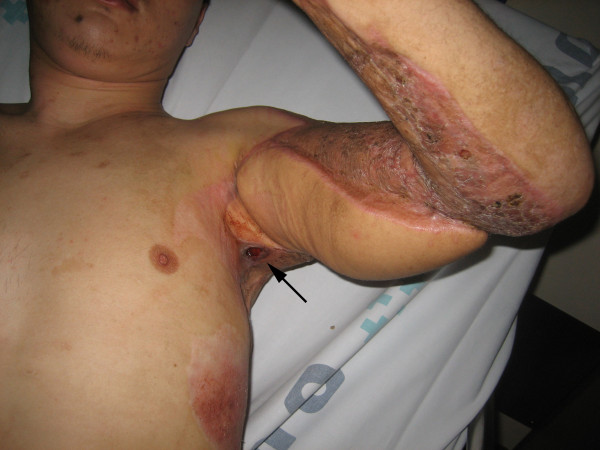
**Initial presentation of the patient.** A round ulcerated wound was noted at the posterior axilla.

**Figure 2:**
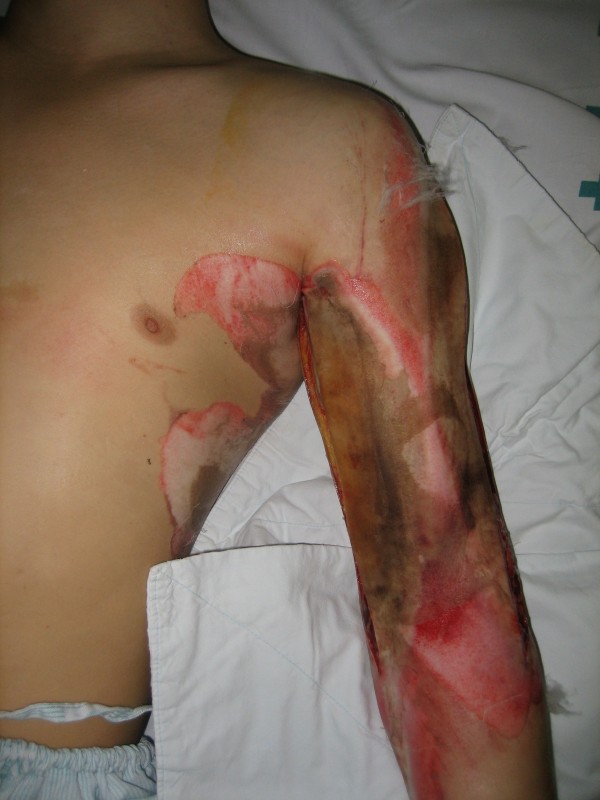
**Clinical image at the time of the contact burn six months earlier.** At the time of the contact burn six months earlier, the patient had undergone immediate fasciotomy for a wound at the medial and lateral aspect of the upper arm. The exposed neurovascular bundle was covered with a latissimus dorsi musculocutaneous flap, and the rest of the lesion was covered with a split-thickness skin graft.

**Figure 3:**
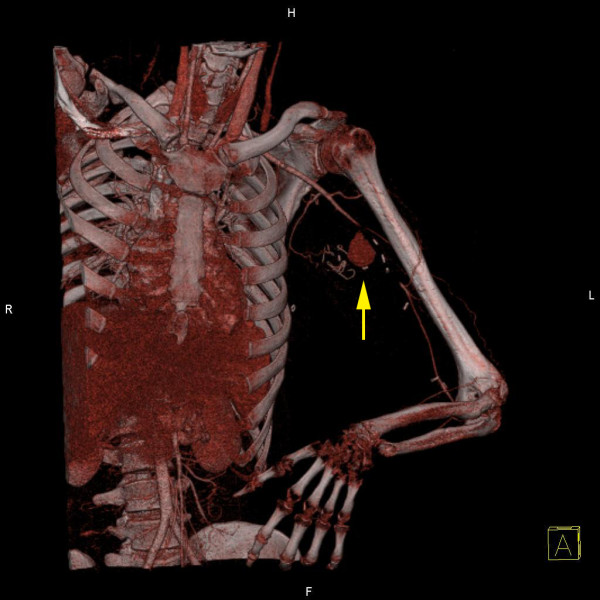
**Preoperative three-dimensionally reconstructed angio CT scan.** Three-dimensionally reconstructed angio CT scan. A pseudoaneurysm in the left brachial artery was noted.

**Figure 4:**
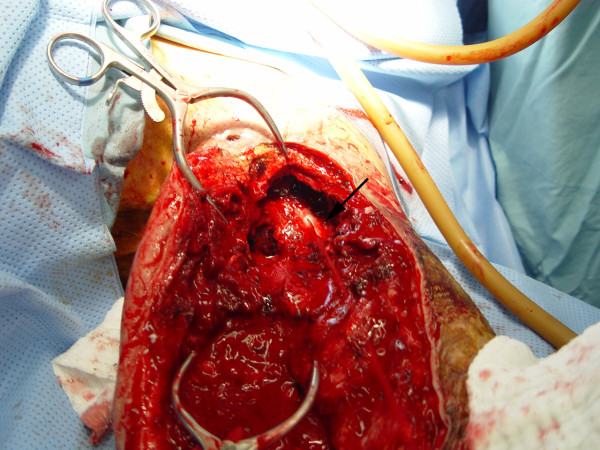
**Intraoperative view.** The aneurysmal sac was removed, and a slit-like defect was noted in the brachial artery, accompanied by blood pumping. Also noted fibrotic adhesions of the neurovascular bundles were evident.

**Figure 5:**
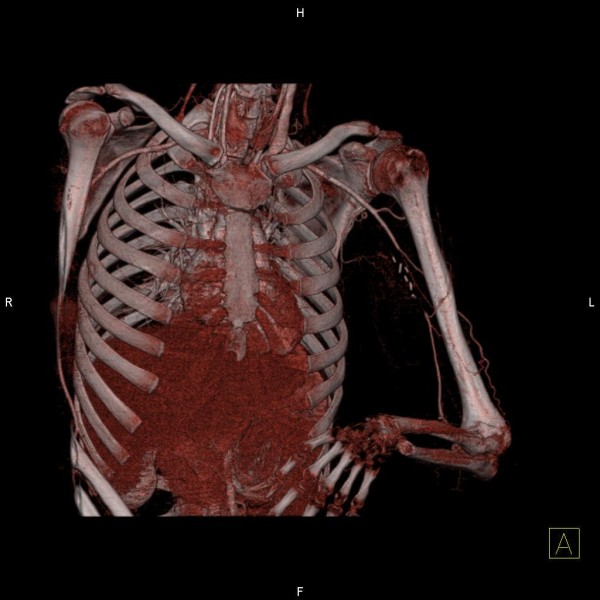
**Ten days postoperative three-dimensionally reconstructed angio CT scan.** Postoperative view of the three-dimensionally reconstructed angio CT scan 10 days after the removal of the pseudoaneurysm. Intact distal flows were noted.

A 26-year old male patient presented to the hospital with wound dehiscence and oozing of the left axilla that had commenced two days earlier while undergoing rehabilitative therapy for postburn joint ankylosis and brachial plexus palsy of the upper extremity (Figure 1). According to the patient’s history, he had undergone escharectomy and latissimus dorsi musculocutaneous flap coverage of a neurovascular bundle exposed in the medial upper arm due to a contact burn of the left upper extremity six months earlier, in addition to a split-thickness skin graft for a lesion (Figure 2). At the time of the hospital visit, the patient’s blood pressure was 130/74 mmHg, and his heart rate was 98 bpm. The hemoglobin value was 12.8 g/dl. The examination revealed no other specific findings. The wound was approximately 1 × 1 cm wide, with bleeding in an oozing pattern. Distal pulsation and circulation had been maintained. Under the assumption that wound dehiscence had occurred during the rehabilitative treatment, a moderate compression gauze dressing was applied. The wound gradually healed, but wound rupture occurred again at the site of the posterior axilla on day 14 of hospitalization. The new site of wound dehiscence was due to a hematoma, which was accompanied by profuse bleeding. A gauze compression bandage was applied again, and a computed tomography angiography (CTA) was conducted. The CTA images revealed a pseudoaneurysm in the brachial artery (Figure 3). Due to the profuse bleeding from wound, the patient’s blood pressure was decreased to 90/50 mmHg, and the heart rate was increased up to 108 bpm. The hemoglobin value was also dropped to 8.2 g/dl. The patient underwent immediate surgical exploration and the pseudoaneurysm was approached through the marginal side of the previously performed latissimus dorsi musculocutaneous flap. The blood-pumping ruptured brachial artery pseudoaneurysm was identified by elevating the flap. The pseudoaneurysm originated from a linear, slit-like longitudinal disruption of the brachial artery (Figure 4). The aneurysmal sac was excised at its base, and the slit-like brachial artery defect was closed with 6-0 Prolene (polyprophylene suture, Ethicon Inc., New Brunswick, NJ, USA) sutures. The brachial artery and accompanying median and musculocutaneous nerves showed fibrotic adhesion to the surrounding muscle and fascia. The tethering adhesions were carefully removed in order to recover neurovascular bundle gliding. The wound was closed with replacing the elevated flap after placing an Jackson-Pratt drain. After the removal of the pseudoaneurysm, the distal circulation was maintained. The patient recieved three packs of packed red blood cells postoperatively and the patient’s vital sign was stabilized again. A CTA taken on postoperative day ten confirmed that the pseudoaneurysm had disappeared and that the distal circulation was being maintained (Figure 5). During one year of postoperative follow up, there was no recurrence of distal circulation impairment or pseudoaneurysms.

## Discussion

An aneurysm is defined as a permanent localized dilatation of an artery with at least a 50% increase in its diameter compared with the expected normal diameter [1]. Aneurysms occurring in the upper extremities can be classified largely into false types and true types. False aneurysms are also known as pseudoaneurysms. They can occur after traumatic penetration of the vessel, causing subsequent hemorrhage and extravasation. The hematoma that forms leads to fibrosis and recanalization of soft tissues. False vessels newly formed in this way resemble true vessels but are characterized by a lining of endothelial cells. True arterial aneurysms are formed when the vessel is damaged, which can enable gradual vasodilation. Unlike the sac shape of true aneurysms, pseudoaneurysms have a uniform shape and form easily at a site of repetitive trauma.

A brachial artery aneurysm usually presents as a pseudoaneurysm and rarely as a true aneurysm [2]. Its etiology can be largely classified into three types: congenital [3]; association with systemic disease, such as Ehlers-Danlos syndrome [4], Menkes disease [5], mycotic aneurysm [6,7], polyarteritis nodosa [8], giant cell arteritis [9], Behcet disease [10], Kawasaki’s disease [11], neurofibromatosis [12], and osteochondroma [13]; and sequelae of trauma, including brachial artery arteriography [14], crutch use [15], humerus fracture [16], supracondylar fracture [17], iatrogenic injury [18], blunt trauma [19], drug abuse [20], and missile injury [21]. The clinical features of brachial artery pseudoaneurysm by etiology are summarized in Table 1.

**Table 1:** A summary of reported etiology of brachial artery aneurysm

**Etiology**	**Sex/Age**	**Onset**	**Symptom & sign**	**Diagnostic modality**	**Treatment**	**Complications**	**Reference**
**True aneurysm**							
Unknown	F/77	2 years ago	Painless swelling	CT angiography	Resection and saphenous vein graft	No	[2]
**Pseudoaneurysm**							
Congenital	M/0	Congenital	Small, non-tender swelling	Arteriography	Resection and end-to-end anastomosis	No	[3]
Association with systemic disease							
Ehlers-Danlos syndrome	M/11	N/A	Pulsating mass	Color-Doppler	Ligation and Excision	N/A	[4]
Menkes disease	F/10m	10 months ago	Pulsating mass	Ultrasonography	Ligation and Excision	No	[5]
Mycotic aneurysm	F/29	9 days after catheterization	Pain, Swelling	Surgical Exploration	Wide excision	Muscle atrophy	[6]
	M/20	26 days after Penicillin injection	Tenderness, swelling, marked induration	Oscillometer and Surgical Exploration	Ligation and Excision	Slight weakness and numbness	[7]
Periarteritis nodosa	F/16w	6 weeks after fever	Bilateral pulsating mass, loss of radial pulse	Arteriography	Prednisone, Observation	No (Size unchanged)	[8]
Giant cell arteritis	M/8	2 months after flu	Pulsatile mass	Surgical Exploration	Resection and saphenous vein graft	No	[9]
Behcet disease	M/18	9 months after oral ulceration	Non-specific, multiple oral ulcer multiple papule on forearm	Angiography	Azathioprine, Prednisolone	Recurred after 18 months later	[10]
Kawasaki’s disease	M/6m	N/A	Axillary mass	Arteriography	Resection and vein graft	No	[11]
Neurofibromatosis	F/30	IUP 34 weeks	Arm pain, enlargement of elbow and forearm	Arteriography	Saphenous vein graft/Transhumeral amputation	Decreased arm function	[12]
Osteochondroma	M/17	4 years ago	Swelling, pain, paresthesia	Surgical exploration	Resection and saphenous vein graft	No	[13]
	M/25	Sudden onset	Pain, swelling, hematoma	Arteriography, Ultrasonography	Resection and saphenous vein graft	No	
Sequela of trauma							
Brachial artery arteriography	M/40-50	2-3 weeks after procedure	Increasing small mass, pulsating mass	Surgical Exploration	Resection and primary repair	No	[14]
Crutch use	M/76	1 year ago	Palpable mass, absent distal arterial pulsation	Surgical Exploration	Resection, saphenous vein and dacron graft	No	[15]
Humerus fracture	M/66	2 months after immobilization	Massively edematous	Arteriography	Shoulder disarticulation	No	[16]
Supracondylar fracture	M/3	7 months after surgery	Slowly growing painless mass	Brachial angiography	Resection and primary repair	No	[17]
Iatrogenic injury	M/56	1 month after venepuncture	Tender, warm, nonpulsatile browny erythematous swelling	Arteriography	Resection and primary repair	No	[18]
Blunt trauma	F/79	16 months after closed reduction	Pain, large lump	Duplex ultrasonography	Resection and arteriorrhaphy	No	[19]
Drug abuse	M.F/32-52	1 day to 4 years	Bleeding hematoma, Painful swelling, Median nerve palsy	Duplex Scan	Resection and Primary repair, Resection and saphenous vein graft	No	[20]
Missile injury	M/14	2 weeks	Tender swelling	CT angiography	Resection and GoreTex patching	No	[21]

Abbreviations: *N/A* Not available, *IUP* Intrauterine pregnancy.

The brachial artery pseudoaneurysm usually develop slowly. It took days to months, even years to develop symptoms or be detected clinically. A brachial artery pseudoaneurysm often presents with erythema and induration, together with an expanding, painful mass. It is sometimes accompanied by a thrill or an audible bruit, decreased temperature, cyanosis, loss of pulsation, and paresthesia upon nerve compression of the distal extremity [22]. Various diagnostic methods can be used, including arterial Doppler ultrasonography, angiography, contrast-enhanced computed tomography (CT), and magnetic resonance imaging (MRI). Although selective arteriography is accepted as the gold standard [23], high-resolution duplex ultrasonography is faster, more cost effective, and more readily available in the emergency department [24].

Very rarely, the presence of a thromboembolism in the aneurysm can result in terminal ischemia, gangrene, and amputation [10]. In such cases, only early diagnosis and treatment can prevent progression to major disability. The treatment of brachial artery pseudoaneurysm depends on the location, size, pathogenesis, and accessibility of the pseudoaneurysm [25]. Surgical methods (ligation, resection and reanastomosis or vein graft interpositioning), endovascular methods (endovascular stent-graft implantation, embolization of sac, embolization of distal and proximal arterial segments), external compression (US-guided), and percutaneous thrombin injection can be used for treatment. Due to the emerging technical evolution of the endovascular intervention, which prevents bleeding and invasive procedure, the need for surgical intervention has decreased. However, there are surgical indications that cannot be substituted with other less invasive methods: rapidly expanding pseudoaneurysm, infected pseudoaneurysm, distal ischemia caused by local pressure by the pseudoaneurysm, neuropathy caused by local pressure, failure of percutaneous treatment, and ischemic soft tissues and skin caused by local pressure [26]. Although a single small pseudoaneurysm that is located distal to the brachial bifurcation can be ligated [25], surgical excision with arterial reconstruction is the standard treatment. The arterial continuity should be restored with end-to-end anastomosis or a venous interposition graft [20,27]. Endovascular stent-grafts implantation is a minimally invasive intervention with a high success rate. However, the high cost of the device, luminal stenosis, and long-term complications, such as device failure, should be considered [28,29]. Embolization of the sac is indicated when the sac is small and the pseudoaneurysm does not disturb the distal circulation. Embolization of the distal and proximal arterial segments is only indicated if collateral circulation is sufficient [25]. US-guided compression was first introduced as a treatment of postangiographic femoral artery injury and also applied for treatment of a brachial artery pseudoaneurysm [30,31]. However, there are limitations, such as a long procedural time, patient discomfort, and lower effectiveness with an anticoagulated patient. When there is infection, coexisting large hematomas with impending compartment syndrome, limb ischemia, skin ischemia, excessive patient discomfort, and unsuitable anatomy, US-guided compression is contraindicated [26]. Percutaneous thrombin injection is performed under US-guide and also conducted with the aid of intraluminal balloon occlusion [32,33]. This has shown a high success rate and a low recurrence and complication rate. However, there have been several reports of complications, such as distal embolization, anaphylaxis, abscess formation, and pseudoaneurysm rupture. There can be complications including median nerve traction due to postoperative adhesion [24], true aneurysm formation [34] and Volkmann’s ischemic contracture [35].

This case did not show the generally observed symptoms of a pseudoaneurysm: swelling, thrill, and a mass-like lesion. A brachial artery pseudoaneurysm was not suspected at first because the patient had visited the hospital with wound dehiscence, accompanied by oozing as the main complaint. It is difficult to perform an accurate physical examination after burn wound reconstruction because the surrounding tissue hardens as a result of fibrosis. This fibrosis of the surrounding tissues also helped to prevent continuous enlargement of the pseudoaneurysm in the present case. The pseudoaneurysm in this patient is likely to have formed gradually due to partial damage of the brachial artery wall during burn rehabilitation when the soft tissues adhered to the blood vessel tract, and due to burn-induced blood vessel injuries. As shown in Figure 4, the pseudoaneurysm originated from a slit-like opening of the brachial artery. And the surrounding neurovascular bundle sheath and muscles had fibrosis as a consequence of the severe burn injury. In a preoperative computed tomography angiography, shown in Figure 3, collateral circulation was noted. Considering the fibrotic surrounding tissue quality and existing collateral circulation, we excised the pseudoaneurysm sac and repaired the slit-like vascular defect with sutures primarily, instead of excision and intervening vascular grafting or bypass grafting after ligation of the brachial artery. Resection and primary repair is one of the usual treatment of brachial artery pseudoaneurysm that is incurred from trauma as shown in Table 1. There was no impairment of the distal circulation and no recurrence of the pseudoaneurysm during the postoperative follow-up period. The nonrecurrence is likely due to the removal of the adhesions around the neurovascular bundle when excising the pseudoaneurysm. However, as adhesion-induced nerve-vessel damage can occur later, a close follow-up is required.

## Conclusions

Delayed rupture of a brachial artery pseudoaneurysm during rehabilitation therapy in a patient with postburn wound reconstruction of the upper extremity is very rare. Nerve-vessel damage may occur in such cases due to adhesion of neurovascular bundle to the surrounding tissues during burn rehabilitation. The exposed neurovascular bundle after fasciotomy in a severe burn patient should be covered with well vascularized soft tissue padding to prevent scarring to the surrounding tissue to prevent scar tethering-induced pseudoaneurysm formation. Although it is hard to observe symptoms of a pseudoaneurysm due to the fibrotic, hard reconstructed tissues, early diagnosis and immediate treatment of the pseudoaneurysm are needed to prevent serious complications, such as distal necrosis.

## Consent

Written informed consent was obtained from the patient for publication of this case report and accompanying images.

## Competing interests

The authors declare that they have no competing interests.

## Authors’ contributions

All of the authors were involved in the preparation of this manuscript. JYL wrote the manuscript and reviewed the literatures. HKim was an assistant surgeon and helped in literature search. HKwon participated in the clinical and surgical management. S-NJ participated in the conception, design of the study, and operated the patient. All authors read and approved the final manuscript.
